# Interactome-Wide Prediction of Protein-Protein Binding Sites Reveals Effects of Protein Sequence Variation in *Arabidopsis thaliana*

**DOI:** 10.1371/journal.pone.0047022

**Published:** 2012-10-15

**Authors:** Felipe Leal Valentim, Frank Neven, Peter Boyen, Aalt D. J. van Dijk

**Affiliations:** 1 Plant Research International, Bioscience, Wageningen, The Netherlands; 2 Hasselt University and Transnational University of Limburg, Hasselt, Belgium; Indian Institute of Science, India

## Abstract

The specificity of protein-protein interactions is encoded in those parts of the sequence that compose the binding interface. Therefore, understanding how changes in protein sequence influence interaction specificity, and possibly the phenotype, requires knowing the location of binding sites in those sequences. However, large-scale detection of protein interfaces remains a challenge. Here, we present a sequence- and interactome-based approach to mine interaction motifs from the recently published *Arabidopsis thaliana* interactome. The resultant proteome-wide predictions are available via www.ab.wur.nl/sliderbio and set the stage for further investigations of protein-protein binding sites. To assess our method, we first show that, by using *a priori* information calculated from protein sequences, such as evolutionary conservation and residue surface accessibility, we improve the performance of interface prediction compared to using only interactome data. Next, we present evidence for the functional importance of the predicted sites, which are under stronger selective pressure than the rest of protein sequence. We also observe a tendency for compensatory mutations in the binding sites of interacting proteins. Subsequently, we interrogated the interactome data to formulate testable hypotheses for the molecular mechanisms underlying effects of protein sequence mutations. Examples include proteins relevant for various developmental processes. Finally, we observed, by analysing pairs of paralogs, a correlation between functional divergence and sequence divergence in interaction sites. This analysis suggests that large-scale prediction of binding sites can cast light on evolutionary processes that shape protein-protein interaction networks.

## Introduction

Genotype-to-phenotype relationships are mediated via molecular networks, including protein-protein interaction networks. Hence, understanding how phenotypes are influenced by sequence changes requires understanding how the specificity of protein interactions is encoded in protein sequences. Identifying which sites are involved in the interactions is a necessary step towards studying the underlying molecular mechanisms and the evolutionary processes influencing protein interaction networks. However, accurate automatic detection of protein binding sites remains a challenge when aiming at large-scale identification.

Those interaction sites composing the protein interface are directly identifiable given a 3D structure of a complex [1]; when only the unbound protein structure is known, predictions based on structural and physicochemical properties [2], [3], [4] are typically used. Although very relevant, protein structure determination is not able to cover the large number of interactions identified by interactome projects [5]. In particular for plants, including the model plant species *Arabidopsis thaliana*, there is a gap between the amount of protein-protein interactions experimentally unravelled and the amount of structural information available in the Protein Data Bank [6]. This gap highlights the need for sequence-based approaches for large-scale predictions of interfaces.

Recently, the Arabidopsis Interactome map has been released, describing about 6,200 highly reliable interactions between about 2,700 proteins [7]. Due to the high rate of gene duplication in the Arabidopsis genome [8], [9], it is particularly interesting to investigate the relationship between protein interaction specificity and sequence diversity in Arabidopsis proteins: after duplication, interaction specificity can diverge causing non-, sub- or neo-functionalization [10]. However, the relationship between interaction specificity and sequence similarity is far from trivial. For example, when analysing pairs of yeast duplicated genes [11] changes in interaction specificity were not correlated with sequence divergence, when this divergence was calculated over the whole length of the protein sequence. Locating the protein-protein binding sites of several duplicated genes may create new routes for this type of investigation, since it would enable to evaluate selective pressure specifically in functional parts of the sequence.

In contrast to protein structures, in which an interaction site is seen as a continuous stretch of amino acids in space, protein sequences show an interface as scattered short sub-sequences. It has been suggested that proteins with common interaction partners also share common functional features [12], such as the short sequences composing the interface. Still, these shared motifs are difficult to discover, perhaps due to their short length. It has also been shown that evolutionary conservation may be useful in predicting functional motifs in the protein surface [13], [14], but for discriminating protein-protein interfaces from other functional sites, e.g. small ligand binding sites and catalytic sites, its use as a stand-alone predictor is questionable [15]. In this work, we evaluate the performance of an interactome-based interaction site predictor when information encoded in the protein sequences is included in its calculation.

We previously developed a method that uses protein-protein interaction networks to find sequence motifs shared by proteins with common interaction partners [16]. This method outperformed existing correlated motif mining algorithms and was able to find biologically meaningful motifs from large protein-protein interaction networks. Here, we present a version of the method modified to account also for the evolutionary conservation of homologous sequences. In addition, the method proposed here restricts the motif search to sequence regions that are likely to be exposed in the protein surface. This new sequence- and interactome-based method predicts motifs that are not only shared by proteins with common interaction partners, but also conserved across sequences of orthologs in closely related species and likely to be exposed in the protein surface.

We start by assessing the performance of our new method. By comparing our predictions against available structural information, we show that the modifications in the method improve its performance. In addition, the assessment provides a basis for determining a set of default parameters for the algorithm. Next, we obtain large-scale predictions of protein interaction sites from the complete Arabidopsis interactome data. We use single nucleotide polymorphism data to obtain evidence that the predicted binding sites are functionally relevant. Subsequently, we analyse available data describing the effect of amino acid mutagenesis to show that our predictions can be interrogated to obtain insight into previously unknown molecular mechanisms underlying the effect of specific mutations. Finally, we analyse the sequences of paralogous pairs to set the stage for further investigations of the molecular mechanisms behind the link between sequence diversity and functional divergence in Arabidopsis proteins.

## Results and Discussion

### SLIDERBio algorithm

**Figure 1:**
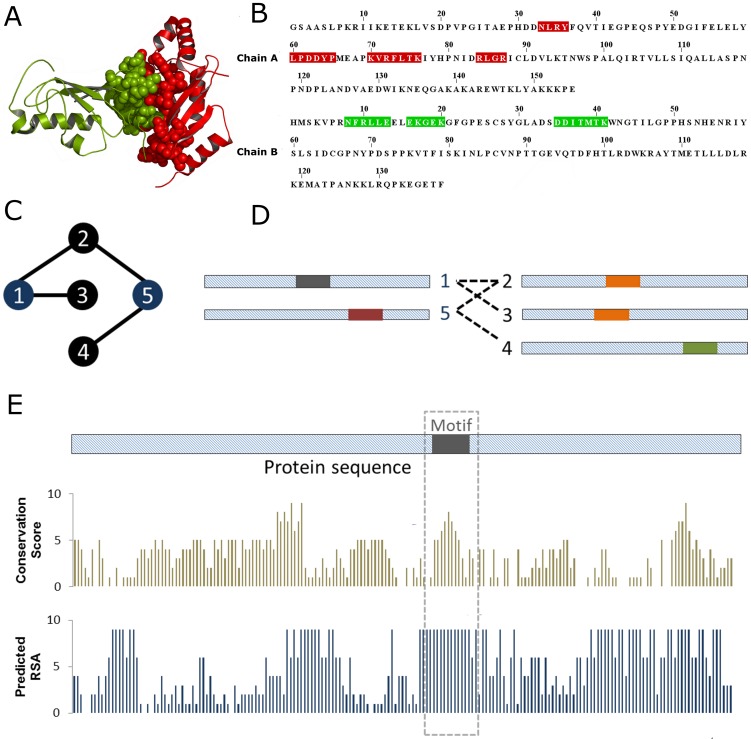
SLIDERBio strategy to predict protein-protein binding sites. (A–B) SLIDERBio follows the assumption that interfaces can be represented by short sequence motifs: (A) Interaction sites (spacefill) are continuous patches of amino acid residues in the 3D structure of a protein, while in a protein sequence (B) the interface is composed of scattered short motifs (regions highlighted in red and green). In (A–B), protein structure and sequence of the Mms2/Ubc13 heterodimer (PDB id 1jat) are used as illustration. (C–D) SLIDERBio predicts interaction sites by finding motif pairs that are overrepresented in pairs of interacting proteins in an interaction network. (C) illustrates a protein-protein interaction network in which the proteins are represented by nodes and the interactions represented by connecting edges; (D) illustrates the protein sequences and their short motifs (regions highlighted in colored bars; same colors represents similar motifs). In this example, the motif pair [grey-orange] is overrepresented compared to the motif pair [red-green]. To calculate the degree of overrepresentation of a motif, the method verifies in how many sequences of interacting proteins a certain motif is found. Originally, SLIDER considered a motif present in a sequence if a perfect match was found between motif sequence and a region in the protein sequence. In contrast, SLIDERBio makes use of a substitution matrix to calculate the similarity between the motif and the sequence. If the degree of similarity between a motif and a sequence is greater than a threshold, SLIDERBio considers that the sequence contains the motif. In addition, SLIDERBio verifies whether the conservation score and the surface accessibility score of the motifs are greater than pre-defined thresholds. These three thresholds are based on the average value per residue over the length of the motif (E).

We recently developed SLIDER, a method that uses a protein interaction network to locate binding sites in the sequence of interacting proteins [16]. To predict binding sites for the proteins in the recently generated Arabidopsis interactome [7], we modified this algorithm to enable it to take various types of biological knowledge into account. Here, we give a brief overview of the method, focusing on the modifications that lead to a novel algorithm. Our method follows the assumption that interfaces can be represented by short sequence motifs (Figure 1). To predict such motifs, the algorithm mines a set of sequences of interacting proteins aiming to find motif pairs overrepresented in pairs of interacting proteins. This mining results in a set of motif pairs that are predicted to be located in protein-protein interfaces. For this work, we extended the original SLIDER algorithm by implementing a different approach to define the presence of a motif in a sequence, and by adding additional filtering steps based on the evolutionary conservation and surface accessibility predicted from the protein sequences. This new, improved version is hereafter named SLIDERBio and is available for download at www.ab.wur.nl/sliderbio.

For computational details of the SLIDER method, the reader is referred to [16]. In summary, the algorithm makes use of an objective function that quantifies the overrepresentation of a motif pair based on its presence in pairs of interacting proteins. To start, it randomly selects a short motif from protein sequences. To optimize the objective function, the algorithm heuristically “slides” the position of the selected motif. This method has been shown to outperform existing methods for mining binding motifs from interaction networks [16].

One critical step in the algorithm consists of verifying whether a short motif is present in a protein sequence. Originally, SLIDER considered that a protein contained a motif if a perfect match was found between motif sequence and a region in the protein sequence. In contrast, the SLIDERBio algorithm makes use of the BLOSUM62 [17] substitution matrix to derive a value that reflects the degree of similarity between the motif and the sequence (see Materials and Methods). In other words, the original SLIDER scanned the protein sequences searching for a perfect match for a motif sequence, while the SLIDERBio algorithm searches for a “close” match. This degree of similarity calculated using the substitution matrix reflects “how close” the match is. Only if the degree of similarity between a motif and a sequence is greater than a threshold, then SLIDERBio considers that the sequence contains the motif.

Additionally, to select only those overrepresented motifs that are likely to be located in the interaction interface, filtering steps based on pre-calculated biological information were implemented. SLIDER considered that a protein contained all the motifs that satisfy the sequence match criteria. For SLIDERBio, the region from the protein sequence that matches the motif has to satisfy two extra conditions: (i) it has to show evolutionary conservation greater than a conservation threshold, and (ii) it has to have predicted surface accessibility greater than an accessibility threshold (Figure 1D). These requirements are based on the fact that interface residues should be located at the surface of a protein (i.e. have high enough accessibility) and that compared to surface residues that are not involved in functions such as protein binding, they are expected to have higher conservation. To implement these filtering steps, the method compares the averages of predicted residue conservation and residue accessibility score calculated over the length of the overrepresented motifs to their thresholds. The strategies to calculate the conservation score and residue surface accessibility are discussed in the Materials and Methods section. Briefly, conservation is assessed using an entropy based score, and residue surface accessibility is predicted using a neural network approach. Values obtained from both approaches are rescaled in the range 0 to 9, and SLIDERBio applies a threshold on those rescaled values. The analysis presented in the section Assessment of SLIDERBio predictions allows determining the best set of threshold values.

Before the modifications, SLIDER required as input only protein sequences and protein-protein interaction data. The SLIDERBio algorithm now additionally requires the conservation score and the predicted surface accessibility for all proteins. In addition, SLIDERBio requires the user to set values for parameters that determine the thresholds of degree of similarity, conservation and residue solvent accessibility. The performance of various parameter settings was analysed by comparing our sequence-based SLIDERBio predictions with available protein structure data. This analysis allowed to assess the significance of the inclusion of the biological information in SLIDERBio and, furthermore, to obtain a default set of parameters. Next, we predicted protein interaction motifs for the Arabidopsis interactome and investigated the predicted interaction sites, in particular aiming at applying these towards understanding the effect of sequence variation.

### Assessment of SLIDERBio predictions

We analysed SLIDERBio predictions aiming (i) to assess the performance of the algorithm towards large scale predictions of protein binding motifs; (ii) to evaluate the significance of the implemented modifications and; (iii) to obtain a set of default values for the parameters. For these investigations, we used a subset of protein-protein interactions such that for the proteins involved, their sequences could be mapped to available structures of protein complexes; hence the interface residues could directly be identified for assessment of our predictions. Hereafter, these subsets are referred to as “structurally mapped datasets”. Although we focus our application on *Arabidopsis thaliana*, for this assessment, given the small number of Arabidopsis proteins with structural mapping, we also used human and yeast protein-protein interaction data (see Figure S1; Tables S1 and S2). We tested SLIDERBio on the structurally mapped datasets of the three species using 180 different parameter settings. To analyse the results, we defined two measures that quantify the quality of the predictions: “Accuracy of predicted motifs” and “Coverage of protein-protein interfaces” (see Materials and Methods). Both measures were combined into an F-score (harmonic mean of Accuracy and Coverage) as overall performance measure.

**Figure 2:**
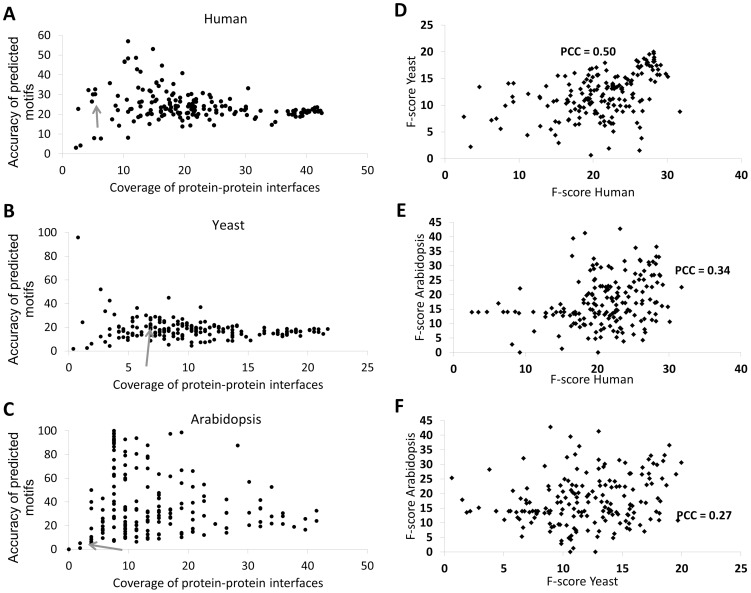
Overall performance of the SLIDERBio algorithm in different datasets. (A–C) Coverage of protein-protein interfaces and Accuracy of predicted motifs. Each dot represents the result of SLIDERBio using one of the 180 tested sets of parameters, for (A) human, (B) yeast and (C) Arabidopsis structurally mapped subsets. The grey arrows indicate the dot corresponding to the result of the previous SLIDER algorithm. (D–F), Correlation of the performance for each of the SLIDERBio parameter settings is compared among datasets of different species: (D) human vs. yeast; (E) human vs. Arabidopsis; and (F) yeast vs. Arabidopsis. Pearson Correlation Coefficient (PCC) is indicated.

Firstly, we observed that for most of the parameter settings, SLIDERBio obtains better results than the previous SLIDER, in terms of both Accuracy and Coverage (Figure 2, A–C). Note that our previous analysis of SLIDER already showed that it obtained improved performance compared to existing correlated motif mining algorithms. Depending on the parameter values, SLIDERBio could predict motifs with Coverage of protein-protein interfaces up to 42%, 22% and 42%, respectively for the human, yeast and Arabidopsis subsets. Likewise, the values of Accuracy of predicted motifs were up to 58%, 96% and 100%. We focus the subsequent analyses based on the F-scores, which give a compromise between ‘Accuracy of predicted motifs’ and ‘Coverage of protein-protein interfaces’.

Secondly, scatter diagrams and Pearson's correlation coefficients (PCC) were used to determine whether F-scores obtained for the same parameter settings are correlated among the three structurally mapped datasets. A strong correlation implies here that the same set of parameters would give results with similar quality in different datasets. A good correlation is particularly important, because we based our assessment on structurally mapped datasets of three species in order to determine the best parameter setting for further predictions on the complete Arabidopsis interactome data. When comparing the F-scores obtained for the same parameters but networks from different species (comparison shown in Figure S1 and S2), we found significant positive correlation: PCC = 0.50, PCC = 0.34 and PCC = 0.27, for correlation of results from human/yeast, human/Arabidopsis and yeast/Arabidopsis, respectively (Figure 2, D–F). From the data in Figure S1 and S2, it is apparent that there is more similarity between the degree distribution of the human and yeast structurally mapped datasets and the complete Arabidopsis interactome than between the Arabidopsis structurally mapped dataset and the complete Arabidopsis interactome. Hence, a reason for the observed smallest correlation between the results in Arabidopsis with those in yeast and human might be that the topology of the structurally mapped Arabidopsis dataset differs most from the other two. In addition, it might also be because of the fact that the number of structurally mapped proteins in the Arabidopsis dataset is much smaller than those of the other species, leading to a larger variation in apparent performance. Overall, the good correlation between the F-scores indicates that parameters that give good results for all three structurally mapped datasets, would also give good results for the complete Arabidopsis interactome.

Thirdly, boxplots were used to group the F-score results according to the used threshold values, thus allowing assessment of the significance of each modification isolated from the effect of the other modifications. The most striking result from this assessment is that, in all the three species, the inclusion of the residue surface accessibility information significantly improved the quality of the results (*p*-value <0.01, paired t-test; Figure S3). Moreover, the highest value of the surface accessibility threshold (value 7) resulted in the highest F-scores, independently of the values that were used for the other two thresholds.

Lastly, we conducted randomization tests to quantify the significance of our results regarding the F-scores, and in addition, to determine the best set of parameters. To obtain *p*-values, we compared the SLIDERBio results against 1,000 sets of randomly generated motif pairs (see Materials and Methods). We selected parameter settings for further consideration using a significance level threshold of *p*-value <0.05 (Figure S4). Note that *a priori* we do not necessarily expect a lot of parameter settings to show significant results, because several parameter combinations will combine biological information in a non-optimal way: e.g. when the threshold for conservation is high and the threshold for accessibility is low, we expect to predict a lot of buried conserved residues instead of interface residues. Although eight parameter settings showed *p*-values less than 0.05 simultaneously for the human and yeast predictions, only one occurred simultaneously for all the three species. Hence, we selected this combination of parameters [Degree of similarity = 0.6; Conservation = 6; Surface accessibility = 7] as the setting to run SLIDERBio for predictions on the full Arabidopsis interactome. These values for the parameters mean that for a motif to occur in a sequence it has to have an average similarity of at least 60%, and that the residue conservation score and residue surface accessibility score have on average values greater than 6 and 7, respectively.

### Protein-protein binding motifs in the Arabidopsis interactome

**Figure 3:**
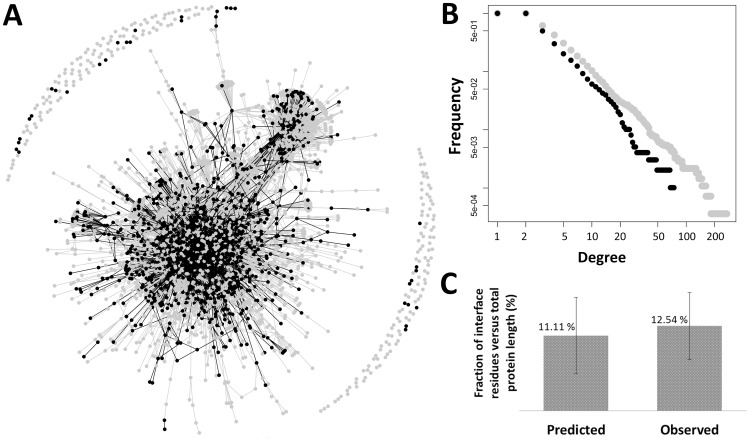
Overall description of the predicted binding sites in the Arabidopsis interactome. (A) Network representation of the Arabidopsis interactome and predicted interaction sites. The vertices and edges in black represent, respectively, the 985 proteins and the 1498 interactions to which predicted motifs are mapped. (B) Degree distributions from the complete protein-protein interaction dataset (grey) and from the subset with only proteins and interactions that have a predicted motif (black). A and B suggest that our method is not biased to predict motifs that can be mapped only to proteins with high degree (*i.e.* number of interactions); moreover, the proteins with predicted motifs are distributed in different positions in the network. (C) Percentage of residues in the interfaces, either in the predicted interfaces or those observed in the structurally mapped dataset. Standard deviation is indicated.

Turning now to the complete Arabidopsis interactome data, our method predicted protein-protein binding motifs that could be mapped (See Materials and Methods) to 1498 (24%) of the interactions among 985 (36%) proteins distributed over the entire network (Figure 3A). Comparison of the degree distribution from the complete dataset against the degree distribution from the subset of proteins with a predicted binding site suggests that the method is not biased to identify motifs only in those proteins with high number of interactions (Figure 3B). Moreover, the motifs mapped onto the protein sequences cover on average 11% of the total protein length, which is a reasonable number given that the equivalent percentage based on protein complexes structures comprising the Arabidopsis structurally mapped dataset is 12% (Figure 3C). For each protein, the resulting predicted sites are given in Table S3; these are also available via www.ab.wur.nl/sliderbio. In addition, for each interaction listed in the interactome, the motif pair(s) predicted to be responsible for the interaction is given. This set of predictions, which is comprised by motifs that are overrepresented in pairs of interacting proteins, conserved across species and predicted to be located in the surface of the protein structure, was used for further analysis.

### Protein-protein binding sites variability and intermolecular coevolution

Conserved residues exposed in the surface of the protein are likely be involved in its biological activity. To obtain an indication of the functional relevance of the predicted binding sites, we used single nucleotide polymorphism (SNP) data (i.e. conservation within *Arabidopsis thaliana*). If our predicted interaction sites are indeed functionally important, one would expect less variability in their positions compared to the rest of the protein sequence. To test this hypothesis, we calculated the percentage of predicted interface residues in which a non-synonymous SNP (nsSNP) is found (1.6%); this is significantly lower than the percentage of all protein residues in which a nsSNP is found (2.2%; *p*-value<0.001; see Materials and Methods). As a control, we tested that a similar signal was not obtained when using synonymous SNPs (data not shown).

Those nsSNP that are found in regions of predicted binding sites are potentially interesting because, by changing protein interaction specificity, they might be responsible for conferring variability to different individuals of a species. However, considering evidence that most interactions are conserved within species [18], one would expect that when an interaction site is mutated, there might be a tendency to have compensating mutations in the interaction partners. Such scenario is consistent with the intermolecular co-evolution model [19]. In our case, it leads to the hypothesis that proteins in which an nsSNP is found overlapping a predicted binding site would be expected to have an increased tendency to interact with other proteins in which an nsSNP is also found in a binding site. To test this hypothesis we counted the number of interactions between proteins in which a nsSNP overlaps a binding site, from which we found a number significantly greater than what would be randomly expected (*p*-value <0.001; see Materials and Methods). This result suggests a tendency for interface residues to co-evolve. Interacting pairs from which both proteins have an nsSNP overlapping a predicted binding site are given in Table S4.

### Putative molecular mechanisms underlying effects of amino acid mutagenesis

A major application of our predictions is to provide sites that can be targeted by mutagenesis to change the interaction specificity of a protein, and to provide putative explanations for observed phenotypic changes upon mutations in terms of changes in interaction specificity. To assess the usefulness of our data towards these goals, we compared our predictions with available results from experimental mutagenesis experiments and their effects on molecular functions and biological processes (see Materials and Methods). The experimentally annotated mutagenesis sites considered here, in general involve residues that are located in functional sites, which in certain number of cases corresponds to protein-protein interaction sites. Hence, one would expect a tendency for the predicted binding sites to coincide with such annotated sites. This was indeed the case: out of 38 proteins for which mutagenesis data is available and for which we predicted the interaction site, for 16 there is at least one mutation site that coincides with a predicted binding site (Table 1).

**Table 1:** Functionally annotated protein sites that coincide with predicted interaction sites.

Protein/Gene name	TAIR/UNIPROT	Amino acids/Mutation	Mutagenesis Effect or Region Annotation	Reference	Predicted site
Acyl-CoA binding protein 5 (ACBP5)	AT5G27630/Q8RWD9	46, 53, 75 and 94/L->Q, Q->A, K->A, F->A	Reduction of oleoyl-CoA-binding	[49]	41 to 48; 51 to 58; 71 to 83; 89 to 94
AFPH2(NINJA)	AT4G28910/Q9SV55	7 to 17	Necessary for the interaction with TOPLESS	[50]	16 to 23
		322 to 425	Necessary for the interaction with the JAZ proteins	[50]	344 to 351; 353 to 360
AtBRE1(HUB1)	AT2G44950/Q8RXD6	712 to 878/Missing in mutant hub1-1/ang4-1	Loss of function	[51]	859 to 869
AtCAND1(CAND1)	AT2G02560/Q8L5Y6	1069/G->D	Reduced response to auxin	[52]	1062 to 1069
CXIP1(GRXS14)	AT3G54900/Q84Y95	133 to 137/SNWPT->AAAAA	Loss of CAX1 activation	[22]	125 to 136
CONSTANS(CO)	AT5G15840/Q39057	96 to 98/Missing in mutant co-1	Late-flowering under long day condition	[53]	93 to 100
IAA3(SHY2)	AT1G04240/Q38822	67 and 69/G->E and P->S	Affects auxin-related developmental processes	[23]	59 to 69
IAA7(AXR2)	AT3G23050/Q38825	87/P->S	Affects auxin-related developmental processes	[54]	77 to 95
IAA19(MSG2)	AT3G15540/O24409	3, 75 and 76/G ->R, P ->L and P ->L	Affects auxin-related developmental processes	[55]	67 to 74
PHABULOSA(ATHB-14)	AT2G34710/O04291	202/G->D	Transformation of abaxial leaf fates into adaxial leaf fates	[56]	198 to 204
TGA1(BZIP47)	AT5G65210/Q39237	260/C->N	Gain of interaction with NPR1	[57]	257 to 264
TIFY 10A(JAZ1)	AT1G19180/Q9LMA8	202 to 228/region missing in mutant jaz1delta3A	Dominant mutation that confers jasmonate insensitivity	[58]	213 to 220
TIFY 6B(JAZ3)	AT3G17860/Q9LVI4	299 to 312/VALPLARKASLARF ->GKKQSQRPDTTFAI	Dominant mutation that confers jasmonate insensitivity	[59]	309 to 318
TOPLESS(TLP)	AT1G15750/Q94AI7	176/K->M	Temperature sensitive gain of function	[60]	171 to 178
YABBY 4(YAB4)	AT1G23420/Q9LDT3	147/K->KLYWSR	Reduced development of the ovule outer integument	[61]	126 to 166
ZEITLUPE(ZTL)	AT5G57360/Q94BT6	200 and 213/L->A, L->A	No ZTL-ASK1 complex formation	[20]	208 to 220

**Figure 4:**
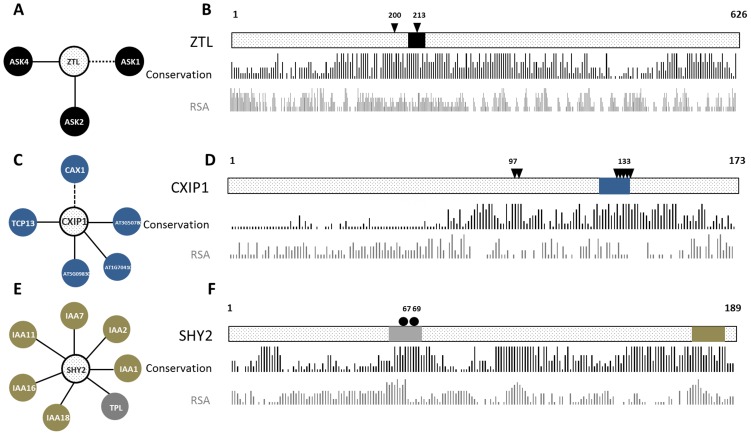
Putative molecular mechanisms underlying effects of amino acid mutagenesis. A, C and E show the interacting partners of the proteins ZTL, CXIP1 and SHY2, respectively (interactions shown as dashed lines are not covered in the Arabidopsis Interactome data). B, D and F show a schematic representation of the sequences of the three proteins, including predicted binding sites (coloured box, using same colour as the proteins predicted to bind to it), mutagenesis sites (triangles for experimental mutagenesis sites, circles for naturally occurring sequence variants) and their positions, and residue surface accessibility (RSA) and conservation (bar plots) as predicted based on the sequence. A–B, in the protein ZTL, alanine mutagenesis of the residues 200 and 213 independently eliminate the interaction with ASK1; for ZTL, the stretch of residues from 208 to 220 is predicted as interaction site for binding with ASK2 and ASK4. This leads to the hypothesis that mutation on ZTP, specifically on the residue Leu213, would not only disrupt its interaction with ASK1, but also with other SKP1-like proteins, such as ASK2 and ASK4. C–D, In CXIP1, alanine mutagenesis of two highly conserved motifs (residues from 133 to 137; and residues from 97 to 100) leads to loss of ability to activate CAX1. For CXIP1, the stretch of residues from 125 to 136 was predicted as binding site, which overlaps the mutated motif SNWPT. The interaction of CXIP1 and the other interacting partners identified in the Arabidopsis interactome, i.e. AT5G09830, AT3G50780, AT1G70410 and TCP13 (AT3G02150), may also be mediated by the same motif. E–F, in the sequence of SHY2, three motifs were predicted as binding sites. The first (residues from 59 to 69; represented in grey) overlaps the position of two naturally occurring mutations (residues 67 and 69) and is predicted to be responsible for binding of TOPLESS (TPL, AT5G27030). A second motif (residues from 180 to 187; represented in brown) is predicted to be responsible for the interactions of SHY2 with six other IAA proteins. This leads to the hypothesis that two known mutations disrupt the interaction of SHY2 with TPL, but the same mutations do not impede its interaction with other IAA proteins.

By analysing details of available annotation for those cases where a predicted binding site coincides with an experimentally annotated mutagenesis site, we found that some of them are indeed involved in protein interactions, whereas for others this is not known but our results provide evidence for such role. For example, in the protein ZEITLUPE (ZTL, AT5G57360), alanine mutagenesis of the residues 200 or 213 located in the F-box domain eliminates the interaction with ASK1 (AT1G75950), in the yeast-two-hybrid system and *in vitro* [20]. Accordingly, for ZEITLUPE, the stretch of residues from 208 to 220 is predicted as interaction site for binding with ASK2 (AT5G42190) and ASK4 (AT1G20140). This leads to the hypothesis that mutation on the F-box domain of ZEITLUPE, specifically in residue Leu213, would not only disrupt its interaction with ASK1, but also with other SKP1-like proteins [21], such as ASK2 and ASK4 (Figure 4, A–B).

A similar case is obtained by analysing available annotation of the protein CXIP1 (GRXS14, AT3G54900), which is thought to activate CAX1 (AT2G38170) through a direct interaction. In CXIP1, alanine mutagenesis of two highly conserved motifs (SNWPT, residues from 133 to 137; and CGFS, residue from 97 to 100) has been shown to lead to loss of ability to activate CAX1, presumably by abolishing the interaction between these two proteins [22]. For CXIP1, we predicted as binding site the stretch of residues from 125 to 136, which overlaps one of the mutation positions. Although CAX1 is not represented in the Arabidopsis interactome data, four other interaction partners for CXIP1 have been identified; i.e. AT5G09830, AT3G50780, AT1G70410 and TCP13 (AT3G02150). We predict that the interaction of CXIP1 with these proteins may also be mediated by the same SNWPT motif (Figure 4, C–D).

Additionally, analysis of available mutagenesis data indicates a number of cases in which mutations are known to affect certain phenotypes, but the molecular mechanism behind this is unknown. Our predictions, together with the Arabidopsis interactome, allow us to generate hypotheses for these unknown mechanisms, which could in principle be experimentally tested. For example, for two naturally occurring mutations in SHY2 (IAA3, AT1G04240) the phenotypic effects have been identified: *shy2-2*, in which a proline in position 69 is mutated to a serine; and *shy2-3*, in which a glycine in position 67 is mutated to a glutamic acid. Although both mutations are known to interfere with auxin-related developmental processes, i.e. root growth, gravitropism and lateral root formation [23], the molecular mechanisms underlying these changes are unknown. In the SHY2 sequence, we predicted as binding site three motifs. One of these, the stretch of residues from 59 to 69, overlaps the position of the two known mutations and is predicted to be responsible for binding of TOPLESS (TPL, AT5G27030). A second motif (residues from 180 to 187) is predicted to be responsible for interaction of SHY2 with six other IAA [24] proteins: IAA1 (AT4G14560), IAA2 (AT3G23030), IAA7 (AT3G23050), IAA11 (AT4G28640), IAA16 (AT3G04730) and IAA18 (AT1G51950). This leads to the hypothesis that mutations in positions 67 and 69 of SHY2 may affect its ability to interact with TOPLESS, but the same mutations do not impede the interaction with other IAA proteins (Figure 4, E–F). Note that the predicted binding site in SHY2 occurs in a region (IAA domain II) which is known to be important for the interaction between IAA proteins and F-box containing proteins [25].

### Gene duplication and protein-protein interaction network evolution

Gene duplication is a major driving force of evolutionary novelty [10]. Because of redundancy immediately after the duplication event, the selective pressure on one of the two copies might be relaxed, both on its *cis*-regulatory elements and its coding sequence. In the latter case, mutations in protein-protein binding sites may either abolish existing interactions or create new interaction sites. These mutations lead to interaction rewiring as one of the mechanisms for functionalization [26]. Here, to assess to which extent mutations in protein-protein binding sites reflect functional divergence, we used our predictions to examine the sequences of 32 paralogous Arabidopsis protein pairs that have previously been classified as having either “no”, “low”, or “high” functional divergence [27] based on examination of knock-out phenotypes.

**Figure 5:**
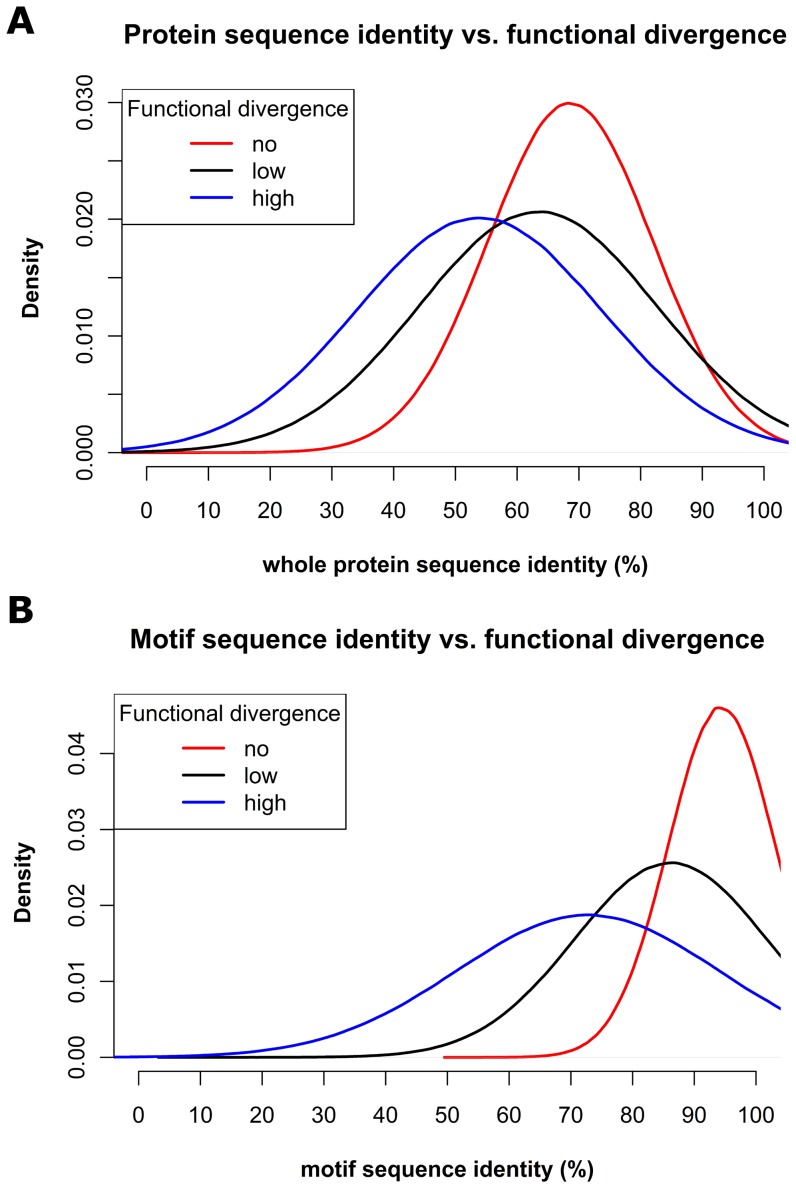
Binding sites contain signal about functional divergence. Distributions of sequence identity values are shown for paralogous pairs classified as having “no” (red), “low” (black) or “high” (blue) functional divergence. The x-axis represents the sequence identity of paralogous pairs. For each paralogous pair, the sequence identity was calculated using either (A) the whole protein sequences, or (B) just the sequence of predicted binding sites. The better separation between the curves for no functional divergence vs. high functional divergence when using predicted interaction sites indicates that these contain signal related to functional divergence.

For the examined paralogous pairs, the sequence identity of the predicted binding sites was better able than the identity of the whole protein sequence to distinguish the three functional divergence groups (Figure 5; Materials and Methods; Table S5). The weak discriminatory power observed by comparing the three density functions for “whole protein sequence identities” (Figure 5A) means that comparing full-length sequence identity gives only a weak indication whether two paralogs are likely to be functionally redundant or functionally divergent. In contrast, the differences among the density functions for the “binding site sequence identities” (Figure 5B) suggests that we may predict the degree of functional divergence based on small sequence changes in the binding sites of paralogous pairs.

The potential for exploiting the sequence of binding sites towards predictions of functional divergence may be illustrated by examining the two paralogs FT (AT1G65480) and TFL1 (AT5G03840). Both genes mediate signals for floral transition in an antagonistic manner: whilst the knockout mutant of FT strongly induces late flowering, the knockout mutant of TFL1 induces early flowering [28]. Based on the overall sequence identity (55%) the pair FT/TFL1 would be classified as non-diverged; however, when using the binding site sequence identity (70%) its most likely classification is “high functional divergence”: the curve for ‘no functional divergence’ has the highest density at 55% for overall sequence identity, but the lowest density at 70% for motif sequence identity (Figure 5). Thus, despite the high overall sequence identity of FT/TFL1, we could correctly infer that the pair shows high functional divergence.

### Concluding remarks

Efficient bioinformatics strategies are crucial to retrieve information encoded in biological networks, in particular to support the formulation of hypotheses on evolutionary processes and molecular mechanisms linking genotype to phenotype. Here, we addressed the challenge of locating, at a large scale, protein binding sites in the Arabidopsis proteins. For this task, we defined a strategy that exploits information encoded in the Arabidopsis interactome and the sequences coding for the interacting proteins. Our sequence- and interactome-based approach enabled the prediction of binding motifs in 985 (36%) of the proteins represented in the interactome. Although this number represents only a small percentage of all Arabidopsis proteins, it is much higher than would be expected from methods that rely on protein structure information. One possible way to achieve higher coverage would be by using a different set of parameters controlling the thresholds of evolutionary conservation and surfaces accessibility of the motifs. Alternatively, predictions based on additional protein-protein interaction datasets [29], [30], [31], [32] could complement the current set of predictions, as will future extensions of the Arabidopsis interactome data. In addition, we recently also developed an extension of the SLIDER algorithm which obtains a much higher coverage of a given network of proteins (Boyen *et al.*, submitted to *Trans Comp Biol Bioinf*) although this does not yet use the biological information sources applied in the current study.

We used our predictions to investigate evolutionary aspects of binding site variability. By assessing the frequency of synonymous and non-synonymous SNPs either in the whole protein sequence or only in the predicted motifs, we found that, overall, our predicted sites are under stronger evolutionary constraints than the rest of the protein. Additionally, we identified non-synonymous SNPs that may be correlated with changes in the protein interaction specificity between different Arabidopsis ecotypes.

Previously, we employed sequence-based approaches [33] to mine binding motifs from the interaction network of transcription factors [29] belonging to the MADS domain protein family [34]. Although the approach used in that work is not applicable to a large interactome due to computational complexity of the algorithm, these results were used to experimentally change the interaction specificity of several MADS domain proteins. This provided insight into mechanisms underlying sub- or neo-functionalization among members of the MADS box family. Here, to corroborate our proteome-scale predictions we used available mutagenesis data (Table 1) to form testable hypotheses for the molecular mechanisms underlying effects of known mutations on several proteins (Figure 4). Our predicted interaction sites are available at www.ab.wur.nl/sliderbio and can be used to pinpoint residues which should be mutated in order to interfere with specific interactions, or to interpret the results of obtained phenotypic changes upon mutations in a molecular and mechanistic way. They also enable to perform large scale studies on the effects of various types of naturally occurring sequence variation on protein interactions, similar to what we recently demonstrated for the MADS domain protein family [35].

It has been debated whether constraints placed on binding sites play a major role in functional divergence [36], when compared to constraints placed on *cis*-elements. Here, Arabidopsis paralogous pairs that have previously been classified, based on morphological changes observed upon mutation, into functional divergence groups [27] were analysed. From our analysis, it seems that the sequence identity calculated over the whole sequence does not contain a lot of signal that explain the observed divergence (Figure 5A). This is in agreement with the findings of [11], in which the correlation between selective pressure on the whole sequence and the functional divergence was assessed. However, when we analysed only the sequence region covered by binding sites (Figure 5B), we found a stronger correlation between functional divergence and selective pressure. Obviously, this does not mean that non-coding sequence divergence (in particular via its effect on gene expression) would not be important for functional divergence, but it demonstrates the importance of coding sequence variation as an additional factor. These examples set the stage for future investigation of the correlation between sequence divergence and phenotypic divergence.

## Materials and Methods

### Protein-protein interactions and sequence data

The *Homo sapiens* (human) and *Saccharomyces cerevisiae* (yeast) protein-protein interaction data used in this work are described in [37]. The *Arabidopsis thaliana* interaction data were obtained from the recently published Arabidopsis interactome map [7]. The sequences of human, yeast and Arabidopsis proteins were retrieved, respectively, from the UniProt [38], Saccharomyces Genome [39] and TAIR [40] databases (see Table S1).

### Mapping protein interacting pairs to known complex structures

One of our assessment procedures aims to verify whether the predicted motifs are located in the protein-protein interface, which is a straightforward task when the structure of the complex is available. However, few complex structures deposited in the PDB correspond to the proteins listed in PPI data used in this work. To overcome such a lack of structural information, we used a strategy to assign sequences to known protein structures based on homology. To link a query sequence to a target sequence with a known 3D structure, we used PSI-BLAST [41] to search against the PDB database under the following conditions: (1) the bit score is higher than 70; (2) the aligned region from the query sequence has a length that corresponds to at least 30% of the query total length; (3) the aligned region from the target sequence has a length that corresponds to at least 30% of the target total length; and (4) the identity of the aligned regions is higher than 40%. Subsequently, we used the sequences and their assigned structures to filter the interacting lists to retain only the interactions for which both proteins link to interacting units of a complex with known structure (e.g. proteins A and B interact, and protein sequence A is assigned to protein structure X chain K, protein sequence B is assigned to protein structure X chain L). The resulting subsets of protein-protein interactions contain for the human, yeast and Arabidopsis, respectively, 539, 263 and 53 interactions among 575, 213 and 67 proteins. We refer to these subsets of the protein-protein interaction networks as structurally mapped datasets (see Table S2).

### Identification of interface residues in protein complex structures

After mapping protein sequences to known structures, the interface residues were identified in the complex structures that were assigned to pairs of interacting proteins. To determine these interface residues, we used NACCESS [42] to calculate the residue solvent accessible surface area for all the complexes and for all the unbound proteins. A residue was classified as interface when the solvent accessible surface area calculated in the complex was smaller than the value calculated in the unbound protein. Following the interface residue identification, the protein sequence was aligned with the sequence of its assigned PDB using Clustal [43] and the alignment was used to map residues from the structure to residues in the sequence. In this way, lists of interface residues and non-interface residues of the interacting proteins comprising the structurally mapped datasets were obtained. This data was used to analyse the performance of the various SLIDERBio parameter settings. Note that as input for SLIDERBio itself, only sequence-based information (conservation and predicted surface accessibility) is used.

### Implementation of conservation, accessibility and similarity matrix in SLIDERBio

We extended the original SLIDER algorithm by adding filtering steps based on evolutionary conservation and surface accessibility as predicted from protein sequences, and by implementing an approach to define the presence of a motif in a sequence based on a substitution matrix. Below, we describe these adjustments to the algorithm.

#### Calculating residue conservation scores

Calculating residue conservation requires three sequential tasks: to select a group of homologous proteins, to align the protein sequence with these homologs, and to quantify the conservation of each residue in the alignment. To select groups of homologs we used OrthoMCL (Version 2.0; [44]) to assign each protein to an OrthoMCL-DB (release 5) group. Next, we used Clustal [43] to align the protein sequence with the sequences of all members of the associated OrthoMCL-DB group. Finally, we used the AL2CO software [45] to obtain a conservation score for each position in the multiple sequence alignments. The AL2CO algorithm performs its calculation in two steps: first amino acid frequencies at each position in the alignment are estimated, and then a score is calculated from these frequencies. We used the methods unweight-frequencies and entropy-based in the first and second step, respectively. To assign a conservation score to each residue in the protein sequence, we used the integer conservation indices resulting from the AL2CO calculation. The AL2CO integer conservation score ranges from 0 to 9, representing low to high conservation, respectively; it is obtained from the entropy-score by a linear scaling (subtracting the minimum value and dividing by the difference between maximum and minimum value) To assess the conservation of a given motif, we use the average of the residue conservation scores over the motif length; only if this average is higher or equal than the conservation threshold, SLIDERBio may consider this motif as a binding site.

#### Calculating residue solvent accessibility scores

The relative solvent accessibility (RSA) of an amino acid residue in a protein indicates its level of solvent exposure. To predict the RSA based on protein sequences, we used the SABLE [46] software that predicts whole residue relative RSA scores from sequences alone using a neural network algorithm trained on PDB structures. SABLE outputs an integer value for each residue, ranging from 0 to 9, representing ‘fully buried’ to ‘fully exposed’, respectively. This output is defined as the ratio of solvent-exposed surface area of a residue to the maximum obtainable value of the solvent-exposed surface area for this amino acid, linearly rescaled in a similar way as described above for the conservation score.

#### Strategy to define motif presence based on substitution matrix

To quantify the overrepresentation of a given motif in the network, our method verifies in how many sequences that motif is present. Instead of searching for perfect matches, SLIDERBio uses a modified version of the BLOSUM62 similarity table to calculate the “degree of similarity” of a given motif for a protein sequence. In this modified similarity table, a perfect amino acid match has value 1, and a non-perfect match has value ranging from 0 to 1 directly proportional to the BLOSUM62 score (this linear scaling is performed for each of the rows of the matrix separately). Our method calculates the residue similarity score and it averages the value over the motif length. Only if this average is greater than or equal to the “degree of similarity” threshold, SLIDERBio considers the motif present in the protein sequence.

### Quality measures for evaluating predictions of protein-protein binding motifs

To assess the quality of the SLIDERBio results, we defined two measures that use the structures of the proteins in the above-mentioned structurally mapped datasets. Here, the ‘Accuracy of predicted motifs’ is defined as the number of motifs correctly predicted to be in the interface as a fraction of all motifs predicted to be in protein–protein interface. A motif is said to be in the interface, if at least one of its residues is identified to be in the interface of its assigned complex structure. The ‘Coverage of protein-protein interfaces’ stands for the number of protein pairs that contain at least one motif mapped to their interface, as fraction of the total number of interacting pairs in the interaction data. Thus, the ‘Accuracy of predicted motifs’ reflects the predictive power of the algorithm toward finding motifs that are indeed located in the interface, and the ‘Coverage of protein-protein interfaces’ reflects its predictive power towards finding motifs explaining the largest number of interactions. The overall performance of the predictions was measured via the F-score, which equals 2*‘Accuracy of predicted motifs’*‘Coverage of protein-protein interfaces’/(‘Accuracy of predicted motifs ’+‘Coverage of protein-protein interfaces’).

### Setting SLIDERBio parameters

For the threshold of the allowed degree of similarity between motif sequence and protein sequence, we tested five different values ([none;0.4;0.5;0.6;0.7], where ‘none’ stands for not having used the modification). For the thresholds of conservation and residue surface accessibility, we tested six different values ([none;3;4;5;6;7]) each. In total, 180 combinations (*5×6×6*) of these values were tested. SLIDERBio predicts a set of *N* motif pairs. For each combination of parameters, we executed SLIDERBio on the structurally mapped datasets for the three species using the following configuration: length of predicted motif *l* = 8; number of allowed wildcard-character *d* = 5; maximum execution time *t* = 60 minutes; number of predicted motif pairs *N* = 1,000. We then mapped the resultant motif pairs in the sequence of pairs of interacting proteins, in such a way that each of the interacting proteins contains one of the motifs in the pair. Subsequently, the ‘Accuracy of predicted motifs’, ‘Coverage of protein-protein interfaces’ and F-score were calculated for all the results. For the analysis of the complete Arabidopsis Interactome, maximum execution time was set to *t* = 24 hours.

### Mapping predicted motif pairs to protein sequences

We used our method to predict motif pairs that are overrepresented in pairs of interacting proteins, conserved across species, and predicted to be exposed in the protein surface. Each motif can usually be “mapped” to more than one protein sequence. This mapping is performed by searching each of the motifs against all the interacting protein sequences; and considering only those matches that fit both requirements for conservation and surface accessibility (i.e. conservation greater than the conservation threshold and surface accessibility greater than the RSA threshold).

### Randomly generated sets of motif pairs

In order to assess the significance of the SLIDERBio results, we created sets of random motif pairs by applying the following strategy: First, we randomly selected a sequence in the input sequence set; next, we randomly sampled from the selected sequence a substring of length *l*, and randomly arranged d wildcard-characters in the substring. The same procedure was repeated to create the second motif in the pair, which resulted in a motif pair. Then, we repeated this step till *N* motif pairs were created. In this way, we created 1,000 sets of *N* motif pairs for each of the structurally mapped datasets (human, yeast and Arabidopsis), using the same set up of parameters controlling the length of the motifs (*l* = 8 and *d* = 5), and the same number of motif pairs (*N* = 1,000).

### Analysis of single nucleotide polymorphism

SNPs were obtained from the currently available 80 accessions from the Arabidopsis 1001 Genome Project [47]. After mapping to protein coding sequences, non-synonymous SNPs were extracted and their positions were compared with positions of predicted interface residues. To compare the significance of the small overlap between non-synonymous SNPs and binding sites, sets of randomly chosen “SNPs” were generated (with the same number of SNPs per protein as in the experimental data) and their overlap with the binding sites was counted (using 1,000 random trials). To compare the significance of the amount of interactions between proteins with SNPs overlapping predicted interaction sites, we randomly selected the same number of proteins from the interactome and counted their number of interactions (using 1,000 random trials).

### Analysis of mutagenesis regions

We retrieved and analysed the field “Experimental info” from the section “Sequence annotation” as deposited in the UniProt database [38]. This describes the effects of mutations of amino acids on the biological properties of proteins. Out of all the 985 protein with interface residues predicted by SLIDERBio, experimental information was available for 38 proteins.

### Gene duplication and Functional divergence analysis

To classify the paralogous pairs as having “no”, “low” or “high” functional divergence, we used data from [27], where the divergence was measured on the basis of morphological consequences observed in null mutants of single genes or pairs of genes. From the obtained list of 492 paralogous pairs, we kept only those pairs from which for at least one of the paralogs interface residues were predicted by SLIDERBio (*n* = 32). Next, we used Needle [48] to compute the global pairwise alignment and to calculate the “whole protein sequence identity” for each pair (see Table S5). Then, we mapped our predicted motifs to the resultant alignments and calculated the “binding site sequence identity” by comparing only the sequence regions to which motifs were mapped. To avoid bias of motifs mapped in regions with long gaps, we removed from the analysis any motifs that were mapped to gapped regions.

For each functional divergence group (“no”,“low” and “high”), we created two density functions by fitting a normal distribution to the calculated values of either “whole protein sequence identity” or “binding site sequence identity”. Prior to the analyses, we tested the normality of each group of values using Lilliefors test for normality with no significant results (*p*-values: (0.5, 0.2, 0.2) and (0.1, 0.4, 0.6); for (“no”,“low” and “high” functional divergence) of “whole protein sequence identity” and “motif sequence identity”, respectively), suggesting that the data is normally distributed.

## Supporting Information

Figure S1 **Topological properties of the protein-protein interaction (PPI) networks and their respective structurally mapped subsets.** To create the basis for comparison and assessment of our predictions, we used the structures of protein complexes in order to identify residues that are located in the protein interface. Because the number of complex structures mapped to Arabidopsis proteins is low, we used two other datasets from which more structures are available; the human and yeast protein-protein interaction networks. (A–C) Graphical representation of the human (A), yeast (B) and Arabidopsis (C) interactome. Nodes represent proteins, edges represent interactions. (D) number of proteins and (E) number of interactions in the PPI datasets. Black, proteins and interactions from which structures could be mapped; grey, complete PPI data.
(TIF)

Figure S2 **Comparison of the topology of the protein-protein interaction networks and their respective structurally mapped subsets.** *x*-axis represents the number of protein partners (degree) and *y*-axis represents the frequency. The Figure allows quantitative comparison of the network composed by the subset of interacting proteins from which structural information is available against the complete set of interactions. By using the degree distributions, we observe that the similarity between the structure mapped subsets for the human and yeast interactomes is high, while the Arabidopsis subset has a quite different degree distribution. In addition, the similarity between the yeast and human structurally mapped datasets and the complete Arabidopsis interactome is higher than the similarity between the Arabidopsis subset and the complete Arabidopsis interactome.
(TIF)

Figure S3 **Assessment of the SLIDERBio performance for different values for the thresholds of Degree of similarity, Conservation and surface accessibility.** The box plots group the F-score results (*y*-axis) based on each used threshold value for the SLIDERBio parameters: (A,B,C) show the results grouped based on threshold values for the Degree of Similarity between motif and protein sequence; (D,E,F) for the Conservation threshold values; and (G,H,I) for the Residue surface accessibility threshold values. The results for the Human, Yeast and Arabidopsis structurally mapped datasets are shown, respectively, in (A,D,G), (B,E,H) and (C,F,I). The boxes labelled as ‘none’ contain the F-score results when SLIDERBio did not use the modification in its calculation. The grey horizontal dashed lines touch the boxes in the group that has given greatest 75^th^ percentile. We then tested whether there is statistical difference in the F-score results when SLIDERBio uses or does not use the modification. The figures show the p-value (P) when the results from the group ‘none’ are compared against the results from the group with greatest F-score 75t^h^ percentile. All *p*-values (*P*) shown in the figures are calculated using a two-tailed paired t-test. At significance level 0.01, we reject the null hypothesis that the means are equal.
(TIF)

Figure S4 **Determination of a default set of SLIDERBio parameter values.** The figure shows the *p*-values calculated by comparing F-scores obtained from the SLIDERBio results against those from random results. *y*-axis represents the p-value; *x*-axis indicates which combination of parameters has been used. For legibility, only results for which the p-value is less than 0.05 are shown. The vertical dashed grey line indicates the single parameter setting that showed *p*-values less than 0.05 simultaneously for all the three structurally mapped dataset. This combination of parameters [Degree of similarity = 0.6; Conservation = 6; Surface accessibility = 7] is used to predict binding motifs on the full Arabidopsis interactome.
(TIF)

Table S1 **Human, yeast and Arabidopsis protein-protein interaction networks used in this work.**
(XLSX)

Table S2 **Structures of protein complexes mapped to sequences of interacting proteins.**
(XLSX)

Table S3 **Predicted interaction motifs for Arabidopsis proteins.**
(XLSX)

Table S4 **List of interacting proteins in which a nsSNP overlaps the binding site of both proteins.**
(XLSX)

Table S5 **Functional divergence classification and sequence similarity analysis of paralogous pairs with predicted motifs.**
(XLSX)
