# Supplementary figures and images for: Interactome-Wide Prediction of Protein-Protein Binding Sites Reveals Effects of Protein Sequence Variation in Arabidopsis thaliana
Source: PLoS One. 2012 Oct 15;7(10):e47022. doi: 10.1371/journal.pone.0047022 (PMC3471968; doi:10.1371/journal.pone.0047022)

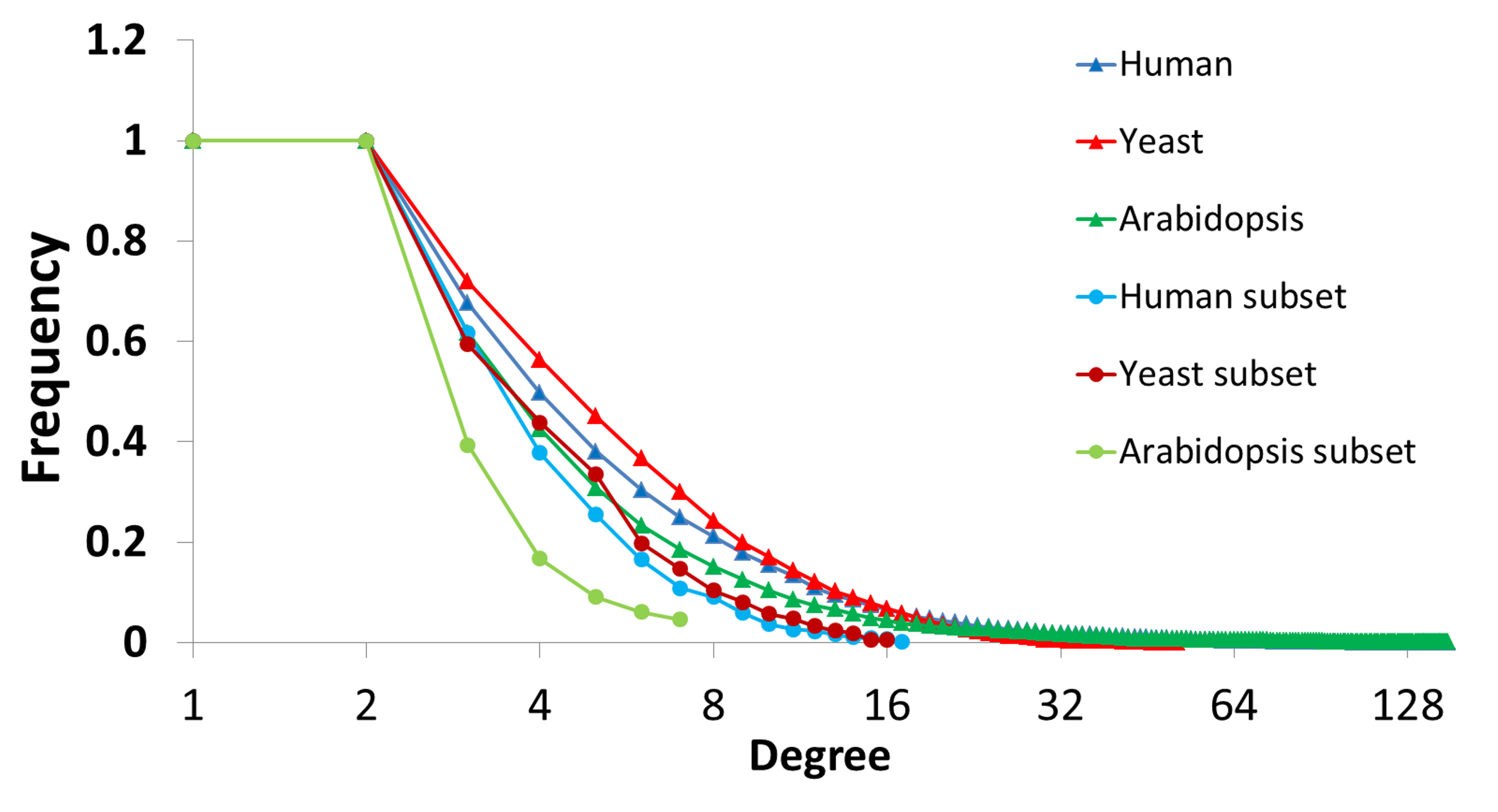

Supplement: Figure S2 — Comparison of the topology of the protein-protein interaction networks and their respective structurally mapped subsets. x-axis represents the number of protein partners (degree) and y-axis represents the frequency. The Figure allows quantitative comparison of the network composed by the subset of interacting proteins from which structural information is available against the complete set of interactions. By using the degree distributions, we observe that the similarity between the structure mapped subsets for the human and yeast interactomes is high, while the Arabidopsis subset has a quite different degree distribution. In addition, the similarity between the yeast and human structurally mapped datasets and the complete Arabidopsis interactome is higher than the similarity between the Arabidopsis subset and the complete Arabidopsis interactome. (TIF) [file pone.0047022.s002.tif]

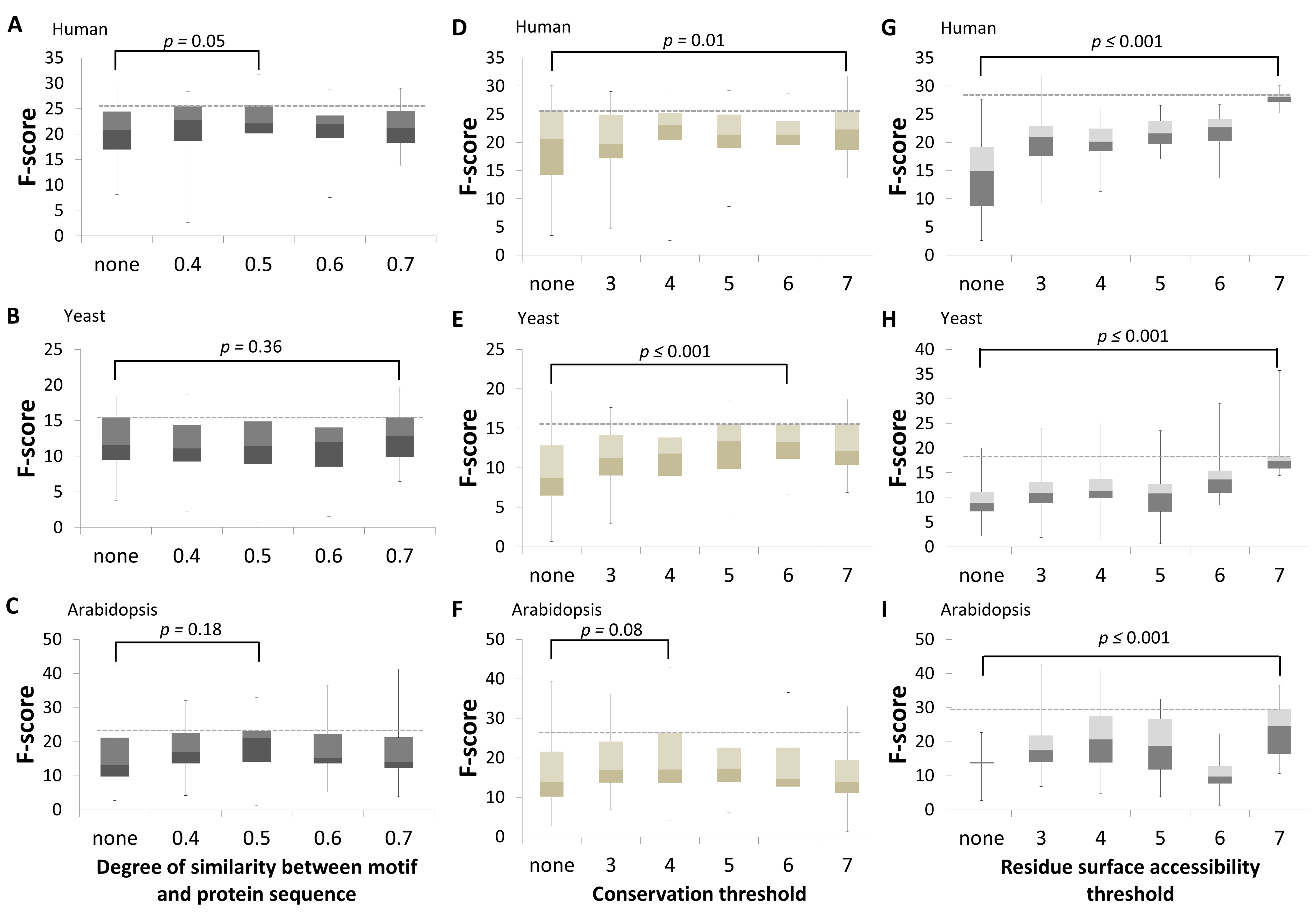

Supplement: Figure S3 — Assessment of the SLIDERBio performance for different values for the thresholds of Degree of similarity, Conservation and surface accessibility. The box plots group the F-score results (y-axis) based on each used threshold value for the SLIDERBio parameters: (A,B,C) show the results grouped based on threshold values for the Degree of Similarity between motif and protein sequence; (D,E,F) for the Conservation threshold values; and (G,H,I) for the Residue surface accessibility threshold values. The results for the Human, Yeast and Arabidopsis structurally mapped datasets are shown, respectively, in (A,D,G), (B,E,H) and (C,F,I). The boxes labelled as ‘none’ contain the F-score results when SLIDERBio did not use the modification in its calculation. The grey horizontal dashed lines touch the boxes in the group that has given greatest 75th percentile. We then tested whether there is statistical difference in the F-score results when SLIDERBio uses or does not use the modification. The figures show the p-value (P) when the results from the group ‘none’ are compared against the results from the group with greatest F-score 75th percentile. All p-values (P) shown in the figures are calculated using a two-tailed paired t-test. At significance level 0.01, we reject the null hypothesis that the means are equal. (TIF) [file pone.0047022.s003.tif]

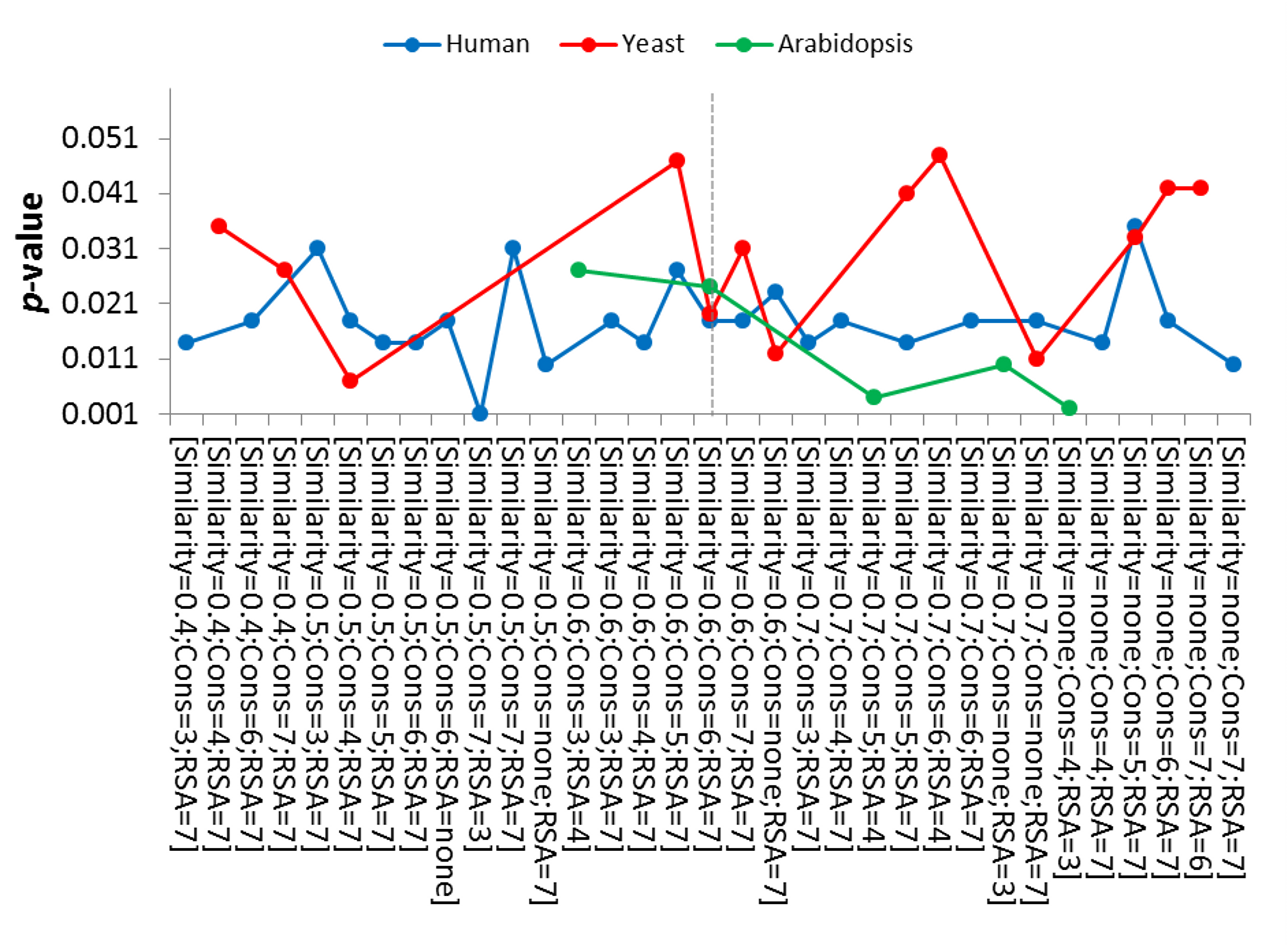

Supplement: Figure S4 — Determination of a default set of SLIDERBio parameter values. The figure shows the p-values calculated by comparing F-scores obtained from the SLIDERBio results against those from random results. y-axis represents the p-value; x-axis indicates which combination of parameters has been used. For legibility, only results for which the p-value is less than 0.05 are shown. The vertical dashed grey line indicates the single parameter setting that showed p-values less than 0.05 simultaneously for all the three structurally mapped dataset. This combination of parameters [Degree of similarity = 0.6; Conservation = 6; Surface accessibility = 7] is used to predict binding motifs on the full Arabidopsis interactome. (TIF) [file pone.0047022.s004.tif]
